# XX/XY Chimerism in Internal Genitalia of a Virilized Heifer

**DOI:** 10.3390/ani12212932

**Published:** 2022-10-26

**Authors:** Izabela Szczerbal, Joanna Nowacka-Woszuk, Monika Stachowiak, Anna Lukomska, Kacper Konieczny, Natalia Tarnogrodzka, Jakub Wozniak, Marek Switonski

**Affiliations:** 1 Department of Genetics and Animal Breeding, Poznan University of Life Sciences, 60-637 Poznan, Poland; 2 Department of Preclinical Sciences and Infectious Diseases, Poznan University of Life Sciences, 60-637 Poznan, Poland; 3 Department of Internal Diseases and Diagnostics, Poznan University of Life Sciences, 60-637 Poznan, Poland

**Keywords:** cattle, disorder of sex development, freemartinism, intersexuality, XX/XY chimerism, *SOX9*, *SRY*, *AMELX*, *AMELY*, *ZFX*, *ZFY*

## Abstract

**Simple Summary:**

Freemartinism is the most common type of disorder of sex development (DSD) in heifers; it is caused by the formation of placental anastomoses between heterosexual twin fetuses and the transfer of masculine factors produced by the testes of the male co-twin to the female fetus. The abnormal development of external genitalia is commonly observed in such heifers, but it cannot be assumed that each heifer with ambiguous genitalia is an example of freemartinism. We genetically analyzed five DSD heifers, and four appeared to be freemartins, as revealed by the presence of XX/XY leukocyte chimerism. The fifth heifer had a normal XX sex chromosome complement and lacked the Y-chromosome-derived genes (*SRY*, *ZFY* and *AMELY*) in blood cells. This heifer was extensively studied through genetic, anatomical, and histological approaches. Postmortem anatomical and histological analysis showed the presence of normal ovaries, oviducts, and uterus, while three Y-linked genes (*SRY*, *ZFY*, and *AMELY*) were detected in DNA isolated from these organs. In conclusion, we suggest that among virilized heifers, there are, besides freemartins with XX/XY leukocyte chimerism, also cases with XX/XY chimerism in internal genitalia, the etiology of which remains unknown.

**Abstract:**

Five DSD heifers underwent genetic analysis in the present study. We cytogenetically analyzed in vitro cultured leukocytes and searched for *SRY*, *AMELX*/*AMELY* and *ZFX*/*ZFY* genes in leukocytes and hair follicles, finding that four of the studied heifers were freemartins (XX/XY leukocyte chimerism). The fifth case had an underdeveloped vulva localized ventrally and cranially to the mammary gland, a normal female sex chromosome complement (60,XX) in the leukocytes, and a lack of Y-chromosome-derived genes in the leukocytes and hair follicles. Postmortem anatomical examination of this heifer revealed the presence of normal ovaries with follicles, uterus, and oviducts, but molecular detection of the *SRY*, *ZFX*, *ZFY,* *AMELX,* and *AMELY* genes in these organs indicated the presence of a cell line carrying the Y chromosome. Further analysis of twelve microsatellite markers revealed the presence of additional variants at six loci in DNA samples derived from the reproductive organs; XX/XY chimerism was thus suspected in these samples. On the basis of the detection of *AMELY* (Y-linked) versus *AMELX* (X-linked) and *SOX9* (autosomal) versus *AMELY* genes by droplet digital PCR (ddPCR), the Y/X and Y/autosome ratios were evaluated; they indicated the presence of XX and XY cell lines in the reproductive tissues. Our study showed that XX/XY chimerism can be present in the internal reproductive organs of the virilized heifers with a normal female set of sex chromosomes (60,XX) and a lack of Y-chromosome-derived genes in the leukocytes. The etiology of this phenomenon remains unknown.

## 1. Introduction

Freemartinism is the most common type of disorder of sex development (DSD) in cattle. It is caused by a transfer of masculine factors from a male fetus to a co-twin female fetus through placental anastomoses. This abnormality is classified as a sex chromosome DSD, and its diagnosis is mainly based on cytogenetic or molecular detection of XX/XY leukocyte chimerism [1,2,3]. Other forms of sex chromosome DSD, such as sex chromosome aneuploidies, have rarely been reported in cattle, while there have been no reports of gene mutations responsible for the DSD phenotype in individuals with the normal complement of sex chromosomes—i.e., XX DSD or XY DSD [4].

The identification of the mechanisms causing DSD phenotype is an important issue from the point of view of breeding. Some DSDs have a de novo origin (e.g., freemartinism, X monosomy, and XXY syndrome) and are not heritable, as the affected animals are sterile. On the contrary, carriers of gene mutations responsible for XX DSD or XY DSD can easily spread the mutation in populations. It is important to point out that distinguishing between heritable and non-heritable DSDs, based on the appearance of external genitalia, is not possible.

In domestic animals, heritable forms of XX DSD are quite common, but it is associated with the presence of ovotestis or testis. Until now, the causative mutation affecting the expression of the *FOXL2* gene involved in ovarian development has been identified only in goats [5,6]. In pigs, the XX DSD is associated with DNA variants in a region harboring the *SOX9* gene, which plays a crucial role in development of the testes [7,8]; in dogs, it is associated with variants near *SOX9* or *PADI6* [9,10,11,12]. Upstream DNA variants of *SOX9* are also known to cause of XX DSD in humans [13].

In cattle, three cases of XX DSD have been reported, and in all these cases, sequences derived from the Y chromosome were detected in the urogenital organs [14], leukocytes [15], blood cells, ovaries, and lymph nodes [16]. However, the *SRY* gene has only been detected in two reports [14,16]. Interestingly, in some DSD heifers, mosaicism with the presence of a triploid cell line carrying the Y chromosome (60,XX/90,XXY) has also been observed (summarized in [17]).

In this study, we analyzed five DSD heifers with ambiguous external genitalia, including a case with extensive virilization. This case was the main subject of molecular analysis due to the presence of a normal set of female sex chromosomes (60,XX) in leukocytes. 

## 2. Material and Methods

### 2.1. Animals

**Figure 1:**
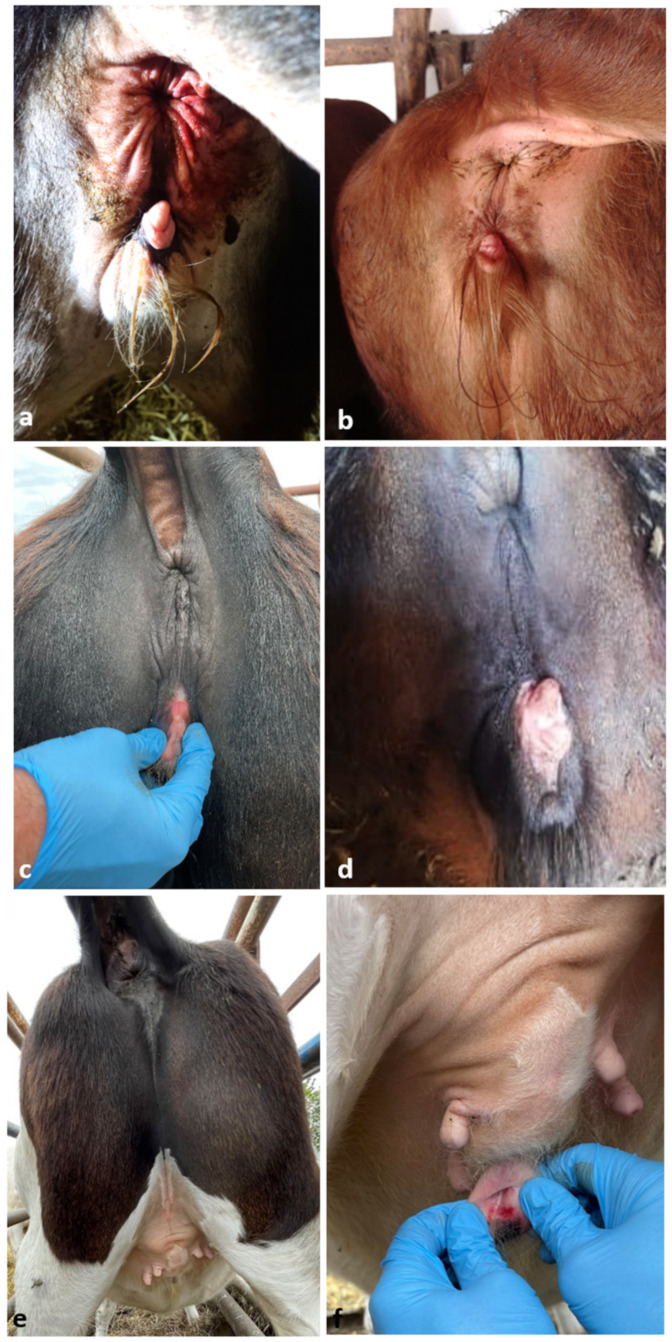
Virilized external genitalia of studied cases. (**a**) Case #7497. (**b**) Case #7502. (**c**) Case #7515. (**d**) Case #7518. (**e**,**f**) Case #7514.

Five heifers (four Holstein Friesians and one Limousin × Simmental crossbred) were subjected to genetic analysis on the request of breeders or veterinarians due to the presence of ambiguous external genitalia (Table 1). These heifers were not related and originated from four farms located in western or central Poland. In four of the heifers, enlarged clitoris or extended anus–vulva distance was observed (Figure 1a–d). The most extensive virilization was observed in the fifth case (#7514), with a rudimentary vulva being ventrally located near mammary gland (Figure 1e,f). 

### 2.2. Histological Studies

Samples of the uterus (approx. 4 cm), oviducts (approx. 1.5 cm), and gonads (approx. 3 and 4 cm) collected postmortem were fixed in neutral buffered 10% formalin solution and used for preparation of paraffin sections (3 µm), which were stained with hematoxylin and eosin (H&E). Microscopic observations were carried out under an Axio Lab.A1 microscope (Carl Zeiss, Oberkochen, Germany) equipped an ERc5s digital camera (Carl Zeiss, Oberkochen, Germany) and analyzed with the use of Zen 2.3 software (blue edition; Carl Zeiss Microscopy, 2011).

### 2.3. Cytogenetic Analysis

Blood samples were collected in heparinized tubes for establishing short-term (48 h) in vitro leukocyte cultures. The cells were cultured in RPMI-1640 medium (Sigma-Aldrich, St. Louis, MO, USA) supplemented with 15% (*v*/*v*) fetal calf serum and 1% (*v*/*v*) penicillin/streptomycin and phytohemagglutinin at 37 °C in a humidified atmosphere of 5% CO_2_. A standard cell culture harvesting procedure was used, including colcemid, hypotonic and fixative treatments. Chromosomes were analyzed using Giemsa staining and C- and G- banding techniques (applied to case #7514), according to methods reviewed by Iannuzzi and Di Berardino [18]. Bovine sex chromosomes were identified based on their biarmed morphology (a large submetacentric X and a small metacentric Y), contrasting with the one-arm morphology of all autosomes, lack of centromeric C band and the characteristic G banding pattern (case #7514). One hundred metaphase Giemsa-stained spreads were analyzed for each case. In addition, twenty C-banded and G-banded spreads derived from DSD heifer #7514 were also evaluated. The slides were examined with an epifluorescence Nikon E600 Eclipse microscope (Melville, NY, USA) equipped with a cooled CCD digital camera (Melville, NY, USA) and Lucia software (Laboratory Imaging, Prague, Czech Republic).

### 2.4. Molecular Detection of X-Linked and Y-Linked Genes

DNA was isolated from blood using DNA Blood Mini kit (A&A Biotechnology, Gdansk. Poland) and from hair follicles using Sherlock AX kit (A&A Biotechnology, Gdansk, Poland). The *SRY* gene fragment covering the whole coding sequence (851 bp) was amplified by PCR using the primers shown in Supplementary Table S1, and its presence was verified using agarose gel electrophoresis. The X-linked and Y-linked (*ZFX* and *ZFY*, respectively) genes were amplified (448 bp) by PCR (Supplementary Table S1) and distinguished by restriction enzyme (*Bsm*I) digestion at 37 °C for 4 h following agarose gel electrophoresis (448 bp for *ZFY*; 391 and 57 bp for *ZFX*). Moreover, PCR detection of the Y-chromosome-derived genes was also performed on DNA samples isolated from the ovaries, uterus, and oviduct (Genomic Mini kit, A&A Biotechnology, Gdansk). All PCR primers were designed using Primer3 (http://www.bioinformatics.nl/cgi-bin/primer3plus/primer3plus.cgi; accessed on 10 August 2009), and all details (primer sequences, annealing temperatures and the amplicon lengths) are shown in Supplementary Table S1.

### 2.5. Analysis of SOX9 and AMELY/AMELX Copy Number

Droplet digital PCR (ddPCR) was used to detect the *AMELX* (X-linked) and *AMELY* (Y-linked) genes, with a fluorescent ratio of *AMELY*/*AMELX* amplicons below 1.0 confirming the presence of XX/XY chimerism, following the procedure described by Szczerbal et al. [3]. Moreover, ddPCR was also used to estimate the copy number of the *SOX9* gene by taking the copy number of the *F2* autosomal gene as a reference [19]. To establish the amplicon ratio of the Y-derived gene (*AMELY*) to the autosomal gene (*SOX9*), an additional reaction was performed with these genes. The procedure described by Nowacka-Woszuk et al. [11] was followed. Briefly, the reaction mixture contained 10 µL of 2×ddPCR Supermix for Probes (Bio-Rad, Hercules, CA, USA), 1 µL of 20× primers/FAM probe, 1 µL of 20× primers/HEX probe, and 1 µL of the *Bsu*I and *Hae*III restriction enzymes for the *AMELX* and *AMELY* genes and the *SOX9* and *F2* genes, respectively. The PCR mixtures were partitioned into approximately 20,000 droplets using a QX200 droplet generator (Bio-Rad, Hercules, CA, USA). PCR was run using following conditions: denaturation at 95 °C for 10 min; 40 cycles at 94 °C for 30 s, and at 55 °C (for *AMELX* and *AMELY*), 57 °C (for *SOX9* and *F2*) and 56 °C (for *AMELY* and *SOX9*) for 60 s (ramp rate 2 °C/s); 98 °C for 10 min, and 10 °C until reading time. The droplets were analyzed on a QX200 droplet reader (Bio-Rad, Hercules, CA, USA). The concentration of the genes was calculated by Poisson distribution using Quantasoft software (Bio-Rad, Hercules, CA, USA). The primer and probe sequences are shown in Supplementary Table S1.

### 2.6. Genotyping of Selected Tissues Using Microsatellite Markers

The genotyping of DSD heifer #7514 was performed using microsatellite (short tandem repeats—STR) markers in DNA samples collected from the blood, hair follicles, ovaries, uterus, and oviduct. Altogether, twelve markers (BM1818, BM1824, BM2113, ETH3, ETH10, ETH225, INRA23, SPS115, TGLA53, TGLA122, TGLA126 and TGLA227) recommended by the International Society of Animal Genetics (ISAG) for parentage testing and genetic profiling were analyzed by a certified laboratory at the Institute of Animal Production (Balice, Poland). Briefly, the analysis was based on the amplified fragment length polymorphisms (AFLP) method, where all markers were amplified in a single multiplex using TypeIt Microsatellite PCR Kit (Qiagen, Hilden, Germany). The amplicons were separated by capillary electrophoresis on Genetic Analyzer 3500 xL (Applied Biosystems, Waltham, MA, USA) with the use of POP-7 polymer (Thermofisher Scientific, Waltham, MA, USA). The length of amplicon was determined using GeneMapper Software 5 (Applied Biosystems, Waltham, MA, USA).

## 3. Results

The microscopic evaluation of cytogenetic slides obtained from in vitro cultured leukocytes could be performed for the four DSD heifers (#7502, #7514, #7515 and #7518), and the molecular detection of the Y-derived sequences could be performed for all DSD heifers (#7497, #7502, #7514, #7515 and #7518).

**Figure 2:**
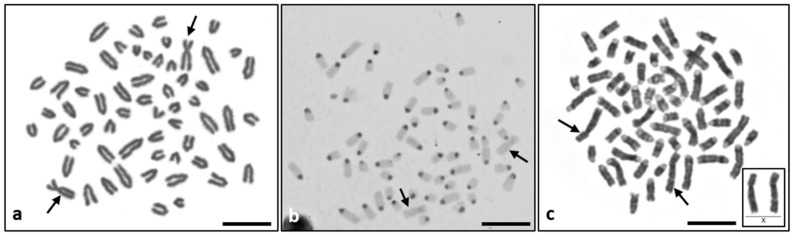
Representative metaphase spreads derived from in vitro leukocyte culture of DSD heifer #7514: (**a**) Giemsa staining—submetacentric X chromosomes are indicated by arrows, (**b**) C-banding—X chromosomes with no centromeric positive C band block are indicated by arrows, (**c**) G-banding—X chromosomes with normal patterns are indicated by arrows and enlarged in a right down corner. Scale bar = 10 µm.

A normal XX sex chromosome complement, analyzed by Giemsa staining, as well as by C- and G-banding (Figure 2) was observed in one of the heifers (#7514), and XX/XY leukocyte chimerism was detected in the other three (#7502, #7515, and #7518) (data not shown). The proportion of XX and XY metaphase spreads varied from XX [98%]/XY [2%] to XX [25%]/XY [75%] (Table 1). In the next step, Y-derived genes (*SRY* and *ZFY*) were not detected in case #7514. On the other hand, both genes were found in blood cells, though not in DNA samples isolated from hair follicles, in the other four cases (#7497, #7502, #7515 and #7518) (Supplementary Figure S1). In addition, the presence of the chimerism was confirmed by estimating the Y/X copy number through ddPCR, based on the number of amplicons derived from the *AMELY* (Y-derived) and *AMELX* (X-linked) genes (Supplementary Figure S2). The results were concordant with those of the cytogenetic analysis. On the basis of these results, case #7514 was tentatively classified as an XX (*SRY*-negative) DSD, while the remaining four cases appeared to be typical freemartins (Table 1).

**Figure 3:**
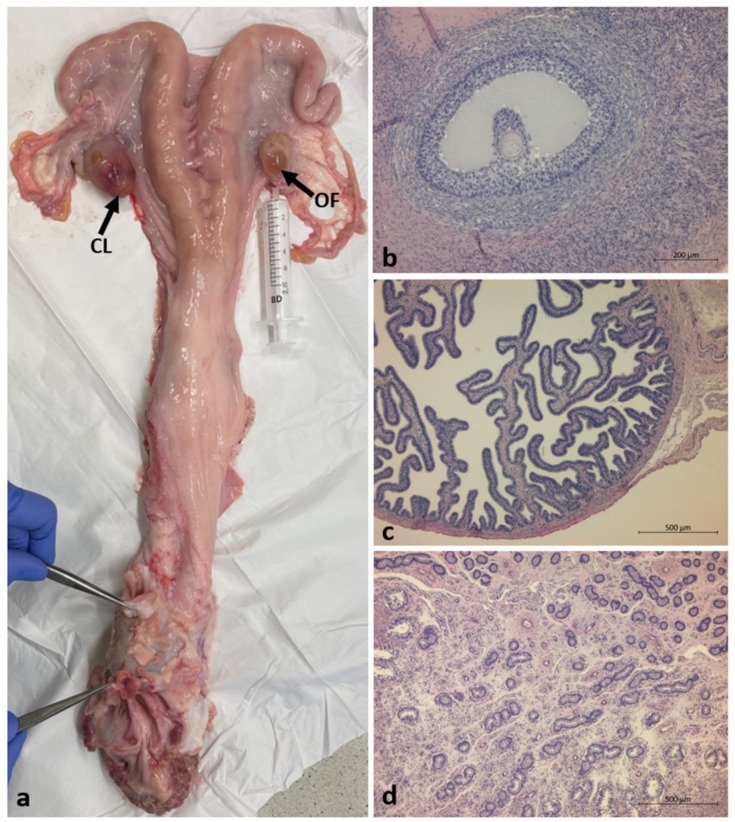
Anatomical and histological analysis of the DSD heifer #7514: (**a**) internal genitalia—uterus with oviducts and ovaries; (**b**) Graafian follicle with an oocyte surrounded by granulosa cells, scale bar = 200 µm; (**c**) cross section of the oviduct: folded mucosa and thin muscularis, scale bar = 500 µm; (**d**) uterine mucosa with small endometrial glands in the basal layer and tubular glands in the functional layer, scale bar = 500 µm. Corpus luteum (CL) and ovarian follicle (OF) are indicated by arrows.

Further analysis focused on DSD heifer #7514. Postmortem anatomical examination of the genitourinary system revealed normal female internal genitalia and virilized external genital organs. The uterine horns and cervix were of normal structure, shape, and consistency (Figure 3a). The ovaries were also normal in structure and shape but contained only a few follicles and corpora lutea. Both oviducts were complete and normal in size. The vagina, despite its normal structure in the cranial part, was dilated in the caudal part. Vulva, vestibule, cervix, uterine body and uterine horns were connected to each other and unobstructed. The absence of a vulval cleft in the perineal area was noted. The urinary bladder and ureter were of normal structure and shape. The urethral orifice was in its normal position in the vagina, and the urethra was connected to the bladder. There was a hypoplastic penile-like structure with a penile retractor muscle connected to the vestibule of the vagina; inside this, there was a virilized urethra with a secondary external orifice located on the ventral body aspect, cranial to the udder. This was in the form of a vulval cleft-like structure or preputial-like structure. The male external genitalia were absent from the inguinal area.

Histological analysis of gonads, oviducts, and uterus showed them to have normal structure (Figure 3b–d). In the ovaries were observed follicles, including a Graafian follicle, as well as corpora lutea and corpora albicans. No structures resembling testicular organization were found.

**Figure 4:**
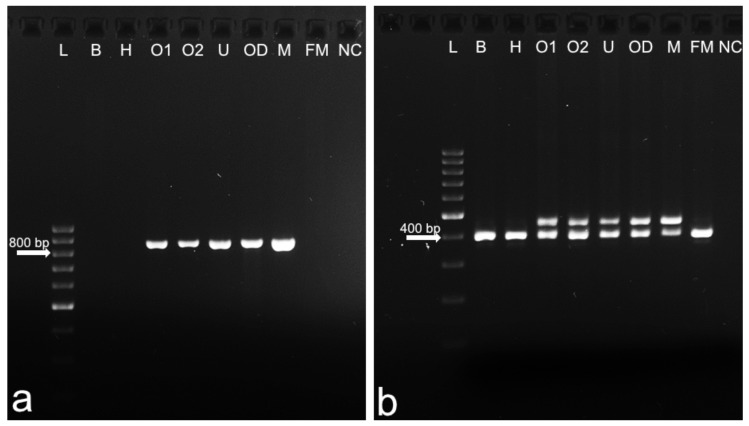
Detection of *SRY* (**a**) and *ZFY* (**b**) genes in DSD heifer #7514. L: GeneRuler DNA ladder; B: blood; H: hair follicles; O1: ovary 1; O2: ovary 2; U: uterus; OD: oviduct; M: control male; FM: control female; NC: negative control (no DNA).

We focused in the first step of the molecular study on elucidating the background of the observed phenotype in DSD heifer #7514. PCR revealed the presence of Y-chromosome-derived genes (*SRY* and *ZFY*) in the gonads, oviduct, and uterus (Figure 4). This observation indicated the presence of another cell line or lines.

**Figure 5:**
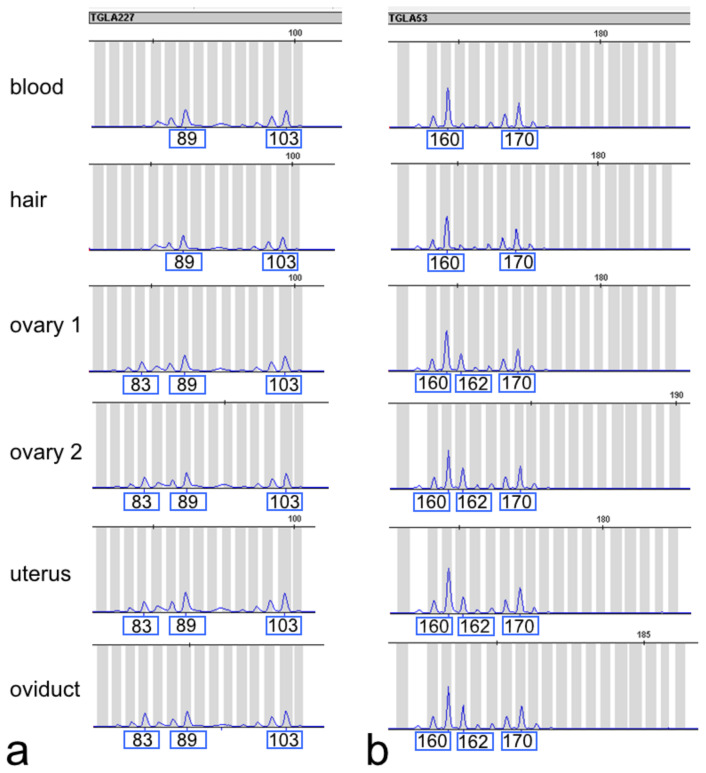
Genotypes for selected microsatellite markers in different tissues of DSD heifer #7514: TGLA227 (**a**) and TGLA53 (**b**). Three size variants for TGLA227 (83, 89 and 103 bp) and TGLA53 (160, 162 and 170) in internal genitalia, instead of two variants observed in blood cells and hair follicles, are visible.

We thus genotyped DNA samples isolated from blood, hair follicles, ovaries, oviduct, and uterus at twelve microsatellite loci, as is commonly done in parentage testing. Additional variants were observed at six loci (ETH3, ETH10, ETH225, SPS115, TGLA53, and TGLA227) in the internal genitalia, while only one or two variants were found in blood and hair follicles (Figure 5; Supplementary Table S2).

**Figure 6:**
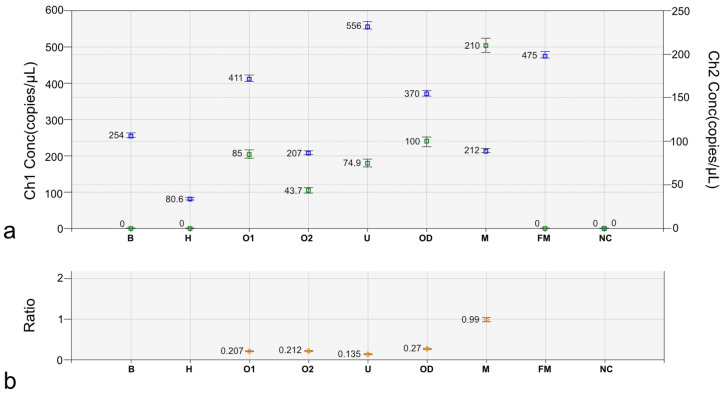
Estimation of the Y/X ratio by ddPCR based on the proportion of *AMELY* (Ch2) and *AMELX* (Ch1) amplicons in the DSD heifer #7514. (**a**) Amplification signals from chromosome X (blue color) and Y (green color). (**b**) Y/X ratio is presented. B: blood; H: hair follicles; O1: ovary 1; O2: ovary 2; U: uterus; OD: oviduct; M: control male; FM: control female; NC: negative control (no DNA).

This result, indicating the presence of chimerism in the internal genitalia, was followed by molecular detection of the number of copies of sex chromosomes and autosomes. Firstly, ddPCR was used to estimate the number of copies of the *SOX9* gene, since an elevated number usually affects gonadal development in females (ovotestis or testis instead of ovaries) and causes virilization. In all studied tissues, the copy number of *SOX9* was normal (two copies), as it was also observed in the studied freemartins (Supplementary Figure S3). Next, ddPCR was used to estimate the Y/X ratio, on the basis of the amplicon numbers of the *AMELY* (Y-linked) and *AMELX* (X-linked) genes. The expected Y/X ratio for a normal male cell line carrying X and Y chromosomes is 1.0, while for a normal female line (XX), it is 0. In our case, the Y/X ratio was low (<0.3, Figure 6), thus confirming the presence of two cell lines (XX and XY). In addition, the *AMELY*/*SOX9* ratio was lowered than the expected 0.5 for a single XY cell line (Supplementary Figure S4). This also indicated the presence of another cell line carrying the Y chromosome in this heifer.

## 4. Discussion

The incidence, consequences, and background of bovine freemartinism have been frequently reported on Esteves et al. [2]. It is well-known that such heifers have underdeveloped internal genitalia and that their external genitalia are often virilized. Heifers born as co-twins to males are usually culled due to the high risk of freemartinism (>90%). However, some freemartins are born as singletons due to early fetal death of the male co-twin [20]. Where virilized genitalia are observed in such heifers, distinguishing between nonheritable freemartinism and other types of DSD requires genetic analysis. Unfortunately, knowledge of the mechanisms responsible for DSD phenotype in heifers with a normal set of XX chromosomes is scarce.

To our best knowledge, there have only been three reports of XX DSD heifers, and in all these cases, Y-chromosome-derived sequences were detected [14,15,16]. In the heifer reported by Takagi et al. [14], a vulval orifice-like structure localized ventrally and cranially to the mammary gland, as well as normal internal genitalia, including ovaries with follicles and corpora lutea, were observed. Interestingly, DSD case #7514 in the present study had a very similar phenotype, and in both cases three Y-linked genes (*SRY*, *ZFY* and *AMELY*) were detected in the internal genitalia, though not in blood cells.

A different XX DSD heifer phenotype was reported by Payan-Carreira et al. [15], who observed rudimentary external genitalia with a small clitoris-like structure, bilateral streak gonads, a normal uterus, a long vagina, and the urethral orifice at the normal location. Fluorescent in situ hybridization (FISH) with a genomic degenerate oligonucleotide-primed (DOP)-PCR probe derived from the heifer revealed the presence of Y chromosome sequences in both X chromosomes, though the *SRY* gene was not detected by PCR.

The third XX DSD case reported in the literature was a female with a normal uterus, ovaries, and mammary gland, but also with a prepuce, normally urinating penis, and scrotum [16]. Molecular analysis revealed the presence of the *SRY* gene in several tissues, including the blood, ovaries, and lymph nodes.

The external genitalia of the DSD heifer (case # 7514) described here were extensively virilized, though a normal uterus and ovaries with follicles were observed. Since earlier reports of XX DSD heifers indicated the presence of Y-derived sequences in some organs, we also searched for three Y-derived genes (*SRY, ZFY* and *AMELY*); we detected them in internal genitalia. Our study thus confirmed that the presence of Y-chromosome-derived genes plays a crucial role in the virilization of XX DSD heifers with female internal genitalia, including ovaries.

It is well-known that the presence of a functional *SRY* gene triggers the development of undifferentiated fetal gonads of mammals toward testis, while ovaries develop when functional SRY transcription factor is not expressed [21]. Thus, the detection of the *SRY* gene in ovaries is a very unusual situation. On the other hand, there are reports suggesting that the XX/XY chimerism can be present in both blood cells and gonadal tissue of bulls originating from heterosexual twins [22,23]. There has also been a report concerning an XY (*SRY*-positive) DSD heifer, in which the *SRY* gene was detected in the blood and ovaries with follicles and a large corpus luteum, despite XX/XY chimerism (freemartinism) being excluded though analysis of in vitro cultured leukocytes and genotyping of eighteen microsatellite markers in DNA isolated from blood [24]. In the present case, we also observed a normal set of female sex chromosomes in leukocytes, and the analysis of microsatellites in DNA isolated from blood cells excluded the presence of the chimerism. In fact, it was in the internal genitalia that the chimerism was detected, as it was revealed by the microsatellite genotyping and ddPCR of sex-linked genes.

## 5. Conclusions

Our study has shown the presence of XX/XY chimerism in internal genitalia, including ovaries, in a DSD heifer with a normal set of female sex chromosomes (XX) in the leukocytes. Since chimersism was not observed in leukocytes, we could exclude a classical form of freemartinism. The most intriguing issue of the presence of the *SRY* gene in normally developed ovaries and the mechanisms responsible for the migration of XY cells to internal female genitalia requires further studies to be elucidated.

## Figures and Tables

**Table 1:** Phenotypes and classification of DSD cases.

Lab No. (Breed *)	External Genitalia	Sex Chromosomes in Leukocytes	*AMELY*/*AMELX* Copy Number Ratio in Blood Cells	Detection of *SRY* and *ZFY* Genes	DSD Classification
7497 (HF)	enlarged clitoris	not analyzed	0.074	present in blood cells, absent in hair follicles	freemartinism
7502 (L × S)	enlarged clitoris	XX [71%]/XY [29%] leukocyte chimerism	0.317	present in blood cells, absent in hair follicles	freemartinism
7515 (HF)	extended anus–vulva distance	XX [98%]/XY [2%] leukocyte chimerism	0.0277	present in blood cells, absent in hair follicles	freemartinism
7518 (HF)	enlarged clitoris, extended anus–vulva distance	XX [25%]/XY [75%] leukocyte chimerism	0.367	present in blood cells, absent in hair follicles	Freemartinism
7514 (HF)	rudimentary vulva localized ventrally, near mammary gland	XX	*AMELY* not detected	present in internal genitalia, absent in blood and hair follicles	XX DSD, with XX/XY chimerism in internal genitalia

* HF: Holstein Friesian; L × S: Limousin × Simmental crossbred.

## Data Availability

The data presented in this study are available on request from the corresponding author.

## Supplementary Materials

The following supporting information can be downloaded at: https://www.mdpi.com/article/10.3390/ani12212932/s1. Supplementary Table S1. PCR conditions applied in molecular analysis of the selected genes. Supplementary Table S2. STR markers analyzed in DSD heifer #7514. Supplementary Figure S1. Detection of *SRY* (a) and *ZFY* (b) genes in freemartin cases. L: GeneRuler DNA ladder; B: blood; H: hair follicles; M: control male; FM: control female; NC: negative control (no DNA). Supplementary Figure S2. Estimation of the Y/X ratio by ddPCR based on the proportion of *AMELY* and *AMELX* amplicons in freemartin cases. (a) amplification signals from chromosome X (blue color) and Y (green color). (b) Y/X ratio is presented. B: blood; H: hair follicles; M: control male; FM: control female; NC: negative control (no DNA). Supplementary Figure S3. *SOX9* copy number by ddPCR for freemartin cases (a) and DSD heifer #7514 (b). B: blood; H: hair follicles; O1: ovary 1; O2: ovary 2; U: uterus; OD: oviduct; FM: control female; NC: negative control (no DNA). Supplementary Figure S4. Estimation of the Y/autosome ratio by ddPCR based on the proportion of *AMELY* and *SOX9* amplicons in the DSD heifer #7514. B: blood; H: hair follicles; O1: ovary 1: O2: ovary 2; U: uterus; OD: oviduct; M: control male; FM: control female; NC: negative control (no DNA).
