# Supplementary material for: XX/XY Chimerism in Internal Genitalia of a Virilized Heifer
Source: Animals (Basel). 2022 Oct 26;12(21):2932. doi: 10.3390/ani12212932 (PMC9659139; doi:10.3390/ani12212932)
Supplement: Supplementary file 1 [file animals-12-02932-s001.zip › animals-1943786-supplementary.pdf]

XX/XY chimerism in internal genitalia of a virilized heifer

by Szczerbal et al.

Supplementary material

Supplementary Table S1. PCR conditions applied in molecular analysis of the selected genes.

Application	Gene	Primers and probe sequences	Amplicon length (bp)	Annealing temperature
PCR	*SRY*	F: 5' aagaacaacttatgaatagcacca<br>R: 5’ ttaagtcgcaggtgaaactgt	851	58°C
	ZFX and *ZFY*	F: 5’ ataatcacatggagagccaccagct<br>R: 5’ gcacttctttggtatctgagaaagt	448	58°C
ddPCR	*AMELX* and *AMELY*	F: 5’ccagccacctgtgc<br>R: 5’ggctgcatggggaata<br>*AMELX*_ probe: 5’cagcgcttgccacc (FAM)<br>*AMELY*_probe: 5’cagcccttgccgc (HEX)	72	55°C
	SOX9 (exon 3)	BIO-RAD Assay ID: dCNS719223989		57°C
	F2 (reference gene)	F: 5’ cctgtctgctgagacgccg<br>R: 5’ gtggtagagttgattctggaatagaaagcat<br>probe 5‘ HEX-ccccgccacccgcagtgtct	96	57°C

Supplementary Table S2. STR markers analyzed in DSD heifer #7514.

Marker	Alleles identified in different tissues					
	blood	hair	ovary 1	ovary 2	uterus	oviduct
BM1818	266/268	266/268	266/268	266/268	266/268	266/268
BM1824	188/188	188/188	188/188	188/188	188/188	188/188
BM2113	127/135	127/135	127/135	127/135	127/135	127/135
ETH3	125/129	125/129	117/125/129	117/125/129	117/125/129	117/125/129
ETH10	219/223	219/223	217/219/223	217/219/223	217/219/223	217/219/223
ETH225	140/148	140/148	140/146/148	140/146/148	140/146/148	140/146/148
INRA23	206/208	206/208	206/208	206/208	206/208	206/208
SPS115	248/248	248/248	248/256	248/256	248/256	248/256
TGLA53	160/170	160/170	160/162/170	160/162/170	160/162/170	160/162/170
TGLA122	147/149	147/149	147/149	147/149	147/149	147/149
TGLA126	115/117	115/117	115/117	115/117	115/117	115/117
TGLA227	89/103	89/103	83/89/103	83/89/103	83/89/103	83/89/103

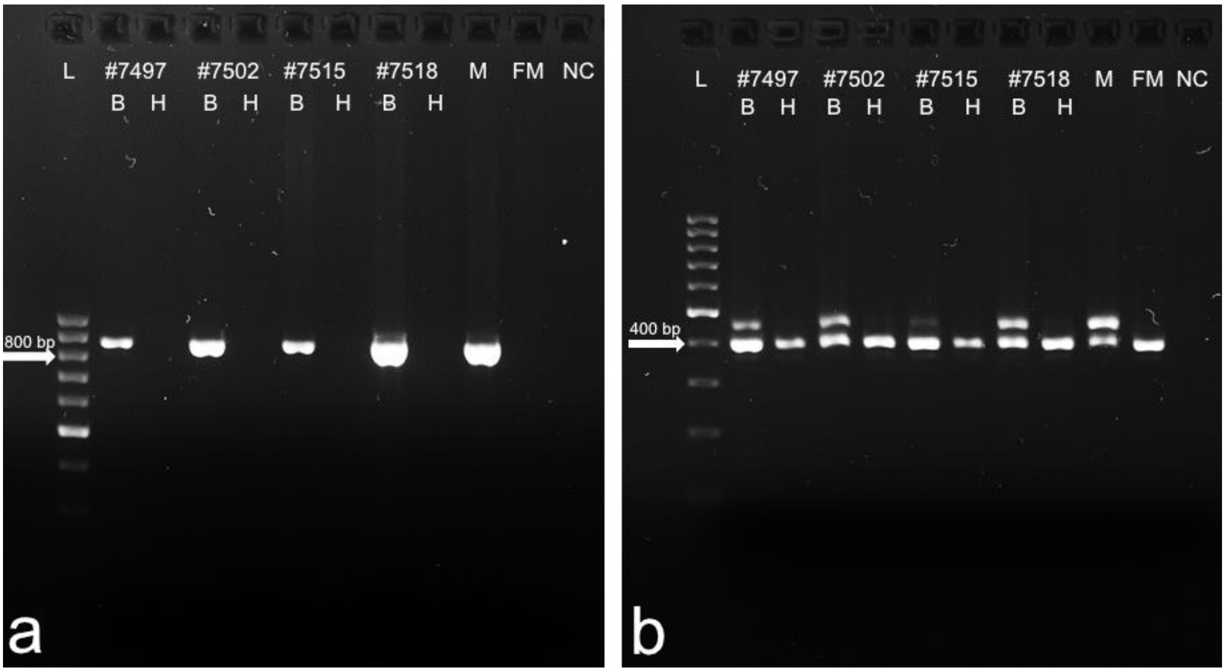

Supplementary Figure S1. Detection of *SRY* (a) and *ZFY* (b) genes in freemartin cases. L: GeneRuler DNA ladder; B: blood; H: hair follicles; M: control male; FM: control female; NC: negative control (no DNA).

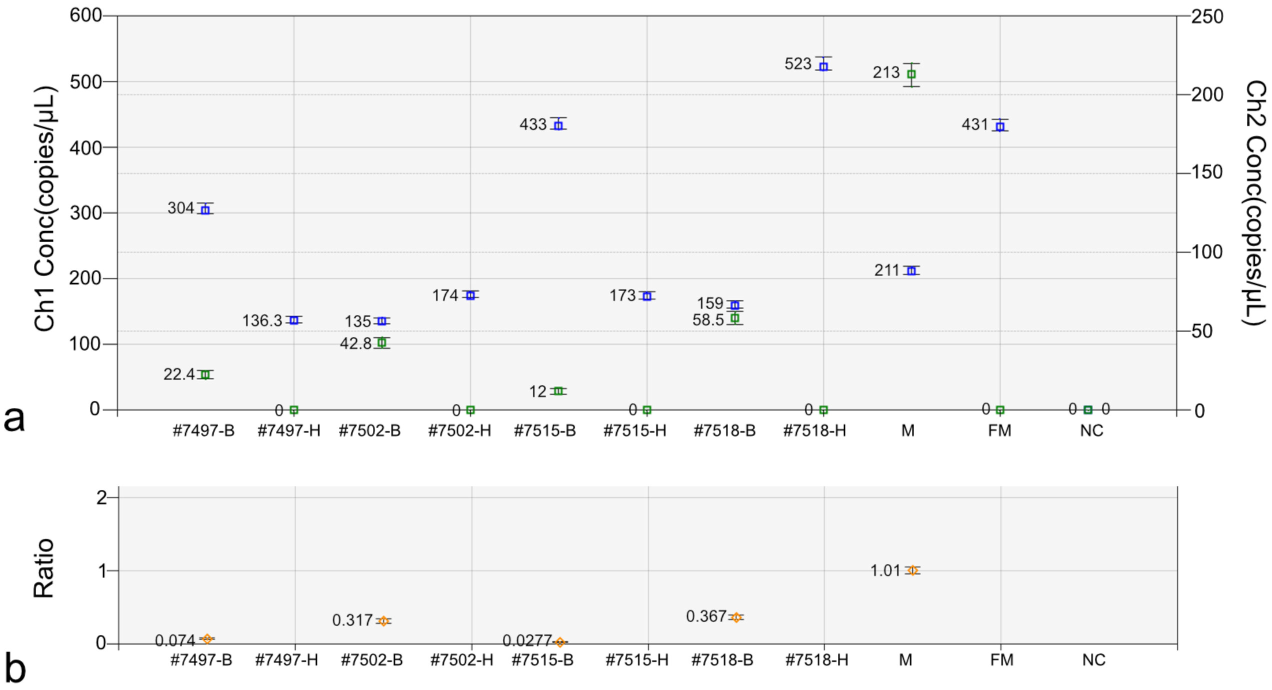

Supplementary Figure S2. Estimation of the Y/X ratio by ddPCR based on the proportion of *AMELY* and *AMELX* amplicons in freemartin cases. (a) amplification signals from chromosome X (blue color) and Y (green color). (b) Y/X ratio is presented. B: blood; H: hair follicles; M: control male; FM: control female; NC: negative control (no DNA).

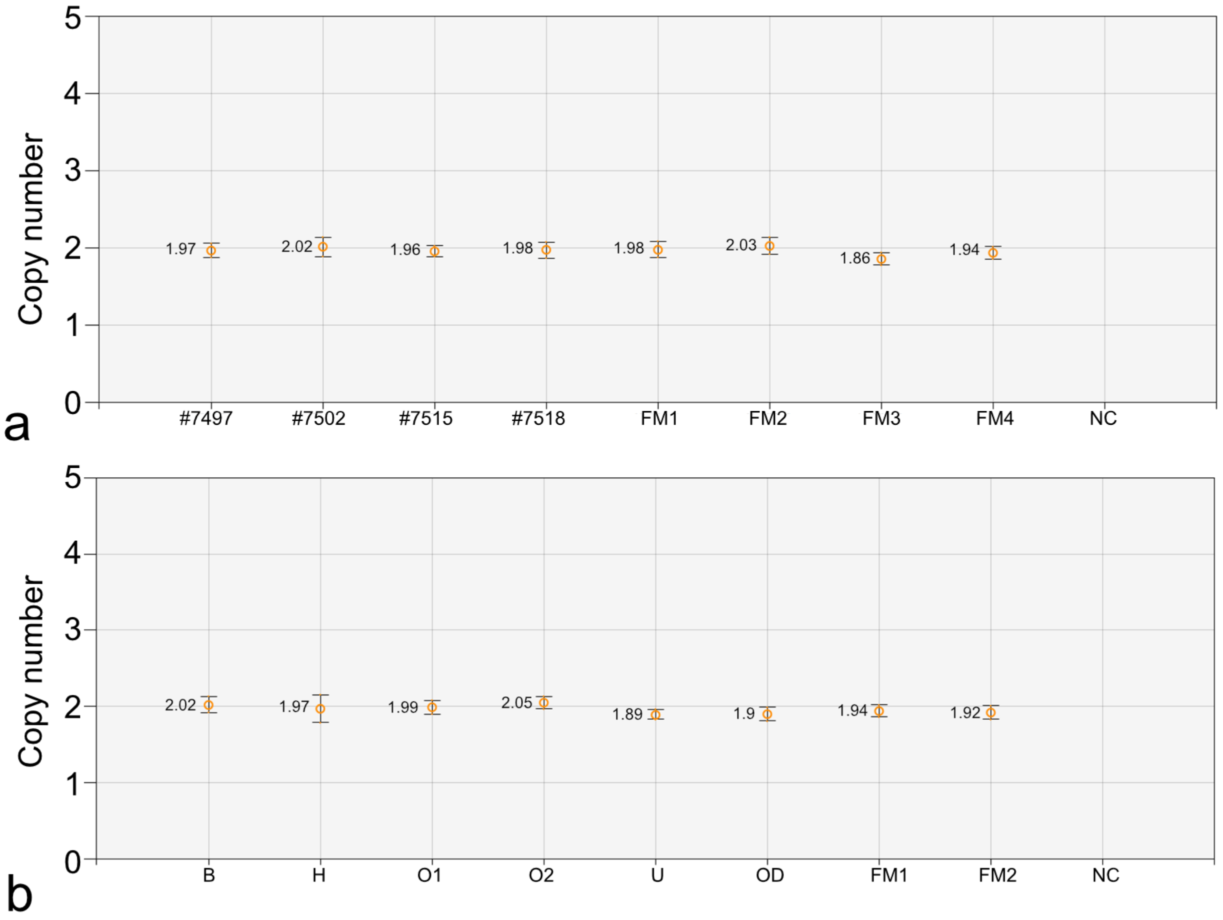

Supplementary Figure S3. Estimation of *SOX9* copy number by ddPCR for freemartin cases (a) and DSD heifer #7514 (b). B: blood; H: hair follicles; O1: ovary 1; O2: ovary 2; U: uterus; OD: oviduct; FM: control female; NC: negative control (no DNA).

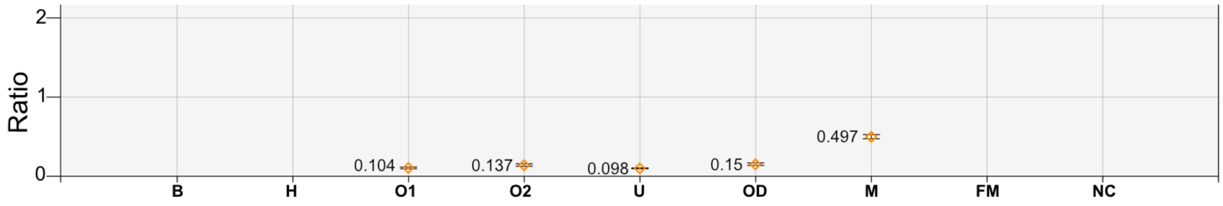

Supplementary Figure S4. Estimation of the Y/autosome ratio by ddPCR based on the proportion of *AMELY* and *SOX9* amplicons in the DSD heifer #7514. B: blood; H: hair follicles; O1: ovary 1: O2: ovary 2; U: uterus; OD: oviduct; M: control male; FM: control female; NC: negative control (no DNA).
